# Racial disparities in access to health care infrastructure across US counties: A geographic information systems analysis

**DOI:** 10.3389/fpubh.2023.897007

**Published:** 2023-04-11

**Authors:** Jingchuan Guo, Sean Dickson, Lucas A. Berenbrok, Shangbin Tang, Utibe R. Essien, Inmaculada Hernandez

**Affiliations:** ^1^Department of Pharmaceutical Outcomes and Policy, University of Florida College of Pharmacy, Gainesville, FL, United States; ^2^West Health Policy Center, Washington, DC, United States; ^3^Department of Pharmacy and Therapeutics, University of Pittsburgh School of Pharmacy, Pittsburgh, PA, United States; ^4^Department of Geology and Environmental Science, Dietrich School of Arts and Sciences, University of Pittsburgh, Pittsburgh, PA, United States; ^5^Division of General Internal Medicine, University of Pittsburgh School of Medicine, Pittsburgh, PA, United States; ^6^School of Pharmacy and Pharmaceutical Science, University of California, San Diego, San Diego, CA, United States

**Keywords:** racial disparities, GIS, health equity, health care infrastructure, health care access

## Abstract

Infrastructure system in the U.S. have been shown to be linked to social and health inequities. We calculated driving distance to the closest health care facility for a representative sample of the U.S. population using ArcGIS Network Analyst and a national transportation dataset, and identified areas where Black residents have a longer driving distance to the closest facility than White residents. Our data demonstrated that racial disparities in access to health care facilities presented large geographic variation. Counties with significant racial disparities were concentrated in the Southeast and did not correspond to counties with a greater proportion of the overall population >5 miles to the closest facility, which were concentrated in the Midwest. This geographic variation demonstrates the need to adopt a spatially explicit data driven approach in the design of equitable health care facility establishment that address the specific limitations of the local infrastructure.

## Introduction

Infrastructure system in the U.S. have been shown to be linked to social and health inequities (1, 2). This has been highlighted by the COVID-19 pandemic, which has caused disproportionate health and economic harm to racial minority groups and socially disadvantaged communities (3). The objective of this study was to calculate driving distance to the closest health care facility for a representative sample of the U.S. population, and identify areas where Black residents have a longer driving distance to the closest facility than White residents.

## Methods

We obtained the addresses of community pharmacies from the National Council for Prescription Drug Programs, addresses of federally qualified health centers from the Health Resources and Services Administration, and of rural health centers and hospital outpatient departments from Centers for Medicare and Medicaid Services. The U.S. population was characterized with the 2010 U.S. Synthetic Population developed by RTI International (4).

For a 1% sample of the synthetic population (*n* = 2,982,544), we computed driving distance to the closest facility using ArcGIS Network Analyst and a national transportation dataset (5). For each county, we calculated the proportion of the population with >5 miles distance to the closest facility, and the odds ratio of having a distance >5 miles to the closest facility for Black compared to White residents.

## Findings

**Figure 1:**
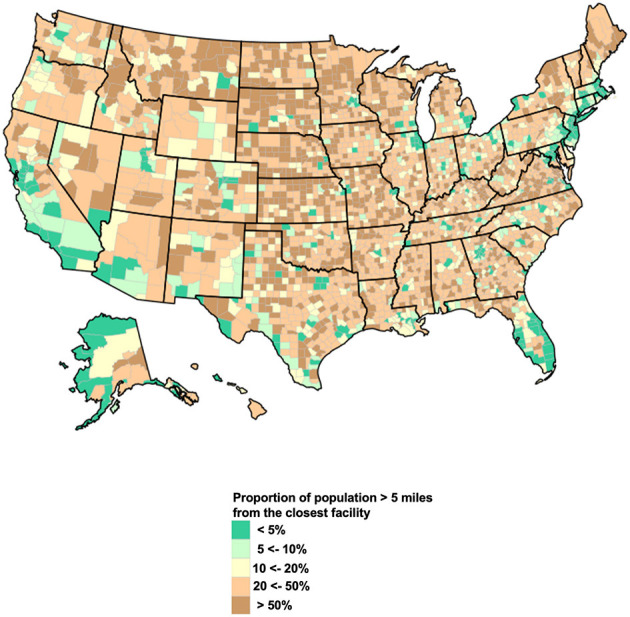
Proportion of population with a driving distance to the closest facility ≥ 5 miles, by county. The map represents the proportion of population in each county with a driving distance >5 miles to the closest health care facility.

The mean (median) number of health care facilities per county was 22 (7). In 889 counties, over 50% of the population had a driving distance >5 miles to the closest facility (Figure 1). These counties were concentrated in the Midwest.

**Figure 2:**
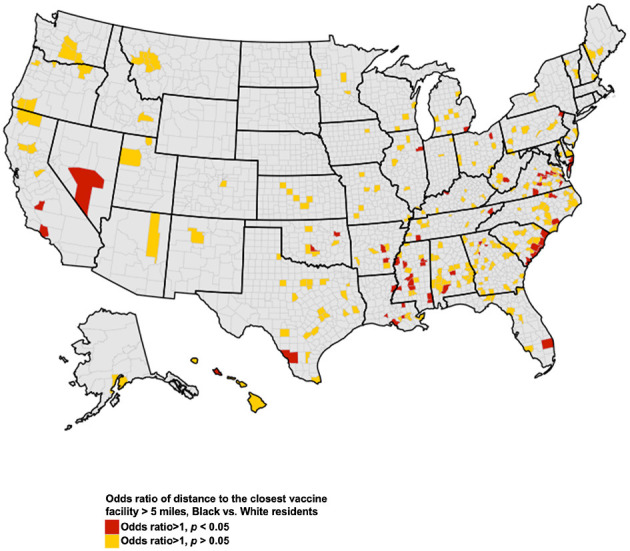
Counties with disparities in access to health care facilities. The map represents counties where Black residents had higher odds of having a driving distance >5 miles to the closest health care administration facility, compared to White residents. Red indicates counties where these disparities were significant at the *p* < 0.05 level.

Black residents were significantly more likely to live >5 miles to the closest facility than White residents in 56 counties (Figure 2). These counties accounted for a total population of 8.3 million and included 18 counties with more than 100,000 residents. The highest concentrations of these counties were in Mississippi (10 counties), Virginia (10), Louisiana (5), South Carolina (5), and Georgia (3). In 233 additional counties, Black residents had higher odds of living >5 miles to the closest facility than White residents, but the difference was not statistically significant. These counties accounted for a total population of 21 million.

## Discussion

Racial disparities in access to health care facilities present large geographic variation. Counties with significant racial disparities were concentrated in the Southeast and did not correspond to counties with a greater proportion of the overall population >5 miles to the closest facility, which were concentrated in the Midwest. This geographic variation demonstrates the need to adopt a spatially explicit data driven approach in the design of equitable health care facility establishment that address the specific limitations of the local infrastructure.

Individuals' socioeconomic status, such as income and education attainment, has been the focus of discussion around barriers to health care access and quality of care among racial and ethnic minority groups, including Black Americans (6). These discussions have often ignored how proximity to healthcare facilities present additional barriers to accessible care. Our geographic information system analysis can guide public health officials to identify areas that necessitate additional infrastructure as well as innovative community partnerships for equitable health infrastructure access. This is of utmost importance to prevent the historical disparities in access to healthcare from further magnifying disparities during public health crisis such as COVID-19 pandemic.

The strengths of the study include nationally representative samples, and the identification of geographic variation in racial disparities in spatial access to health care facilities. Nevertheless, our study is subject to limitations. Non-significant disparities are presented because our 1% sampling of the US population may have resulted in under-power to detect disparities among nonmetropolitan counties at the statistical significance level. Due to lack of ethnicity data in the U.S. synthetic population, it was not possible to estimate access for Hispanic residents.

Our data demonstrates the structural inequities in access to the existing health care infrastructure across racial groups. These inequities should be addressed through the establishment of high-quality health care facilities in under-resourced communities, the expansion of public transportation, and improved community partnerships.

## Data availability statement

The original contributions presented in the study are included in the article/supplementary material, further inquiries can be directed to the corresponding author.

## Author contributions

JG: conceptualization, formal analysis, investigation, methodology, visualization, and writing—original draft. IH and LB: conceptualization, investigation, methodology, funding acquisition, supervision, resources, and writing—review and editing. SD, ST, and UE: investigation, methodology, writing—review, and editing. All authors contributed to the article and approved the submitted version.

## References

[1] Jones SH Armanios DE. Methodological framework and feasibility study to assess social equity impacts of the built environment. J Construct Eng Manag. (2020) 146:11. 10.1061/(ASCE)CO.1943-7862.000191429515898

[2] National Partnership for Women and Families. Advancing Health Equity: Addressing the Role of Structural Racism. (2021). Available online at: https://www.nationalpartnership.org/our-work/resources/health-care/advancing-health-equity-addressing-the-role-of-structural-racism.pdf (accessed June 01, 2021).

[3] Reyes V. The disproportional impact of COVID-19 on African Americans. Health Human Rights. (2020) 22:299−307. 33390715 PMC7762908

[4] International R. U.S. Synthetic Household Population™ Database. (2021). Available online at: https://synthpopviewer.rti.org/obesity/downloads/rti_synthpop.pdf (accessed June 01, 2021).

[5] National Transportation Dataset (NTD). (2021). Available online at: https://www.sciencebase.gov/catalog/item/4f70b1f4e4b058caae3f8e16 (accessed June 01, 2021).

[6] Shavers V. Measurement of socioeconomic status in health disparities research. J Natl Med Assoc. (2007) 99:1013–23. 17913111 PMC2575866

